# Gut Microbiome Alteration after Reboxetine Administration in Type-1 Diabetic Rats

**DOI:** 10.3390/microorganisms9091948

**Published:** 2021-09-14

**Authors:** Sinem Aydin, Ceren Ozkul, Nazlı Turan Yucel, Hulya Karaca

**Affiliations:** 1 Department of Pharmaceutical Microbiology, Faculty of Pharmacy, Anadolu University, Eskisehir 26471, Turkey; sinemtekinkoca@gmail.com.tr; 2 Department of Pharmaceutical Microbiology, Faculty of Pharmacy, Hacettepe University, Ankara 06230, Turkey; cerenozkul@hacettepe.edu.tr; 3 Department of Pharmacology, Faculty of Pharmacy, Anadolu University, Eskisehir 26471, Turkey; nazlituran@anadolu.edu.tr

**Keywords:** gut microbiome, antidepressant, reboxetine, type-1 diabetes

## Abstract

Antidepressants are drugs commonly used in clinical settings. However, there are very limited studies on the effects of these drugs on the gut microbiota. Herein, we evaluated the effect of reboxetine (RBX), a selective norepinephrine (noradrenaline) reuptake inhibitor (NRI), on gut microbiota in both diabetic and non-diabetic rats. This is the first report of relation between reboxetine use and the gut microbiota to our knowledge. In this study, type-1 diabetes induced by using streptozotocin (STZ) and RBX was administered to diabetic rats and healthy controls for 14 days. At the end of the treatment, stool samples were collected. Following DNA extraction, amplicon libraries for the V3-V4 region were prepared and sequenced with the Illumina Miseq platform. QIIME was used for preprocessing and analysis of the data. As a result, RBX had a significant effect on gut microbiota structure and composition in diabetic and healthy rats. For example, RBX exposure had a pronounced microbial signature in both groups, with a low Firmicutes/Bacteroidetes ratio and low *Lactobacillus* levels. While another abundance phylum after exposure to RBX was Proteabacteria, other notable taxa in the diabetic group included Flavobacterium, Desulfovibrionaceae, Helicobacteriaceae, Campylobacterales, and Pasteurellacae when compared to the untreated group.

## 1. Introduction

Major depression (MD) is a significant but treatable disorder prevalent worldwide and can cause many secondary diseases. Sometimes, it is a secondary condition alongside various primary illnesses. Studies show that one in five individuals surveyed have suffered from this common illness in their lifetime and received antidepressant therapy, and 51% of patients with chronic diabetes and heart failure had symptoms of depression [1,2,3,4].

Several drug treatment options are available for major depression, but RBX was the first commercially available selective norepinephrine (noradrenaline) reuptake inhibitor used for treatment since 1997 in many European countries [5,6,7]. RBX inhibits extracellular norepinephrine (NE) reuptake by binding to the norepinephrine transporter molecule. Thanks to the remarkable selectivity of RBX with negligible effects on the serotonin (5-HT) transporters (monoamine, histamine, and acetylcholine), it has shown fewer cardiovascular, gastrointestinal, and urogenital side effects compared with other NRI drugs [8] Recent studies demonstrate that when evaluating drug side effects, we should no longer disregard the microbiome. The adverse effects of antibiotics on gut microbiome composition have long been under scrutiny. Many recent studies show that drugs including antidiabetics, proton pump inhibitors, nonsteroidal anti-inflammatory drugs, and antipsychotics can cause adverse changes to the human body, especially in the gut microbiome composition, which is crucial for human health. However, studies on the alteration of the microbial composition of the gut by intake of antidepressants are limited [9,10,11,12,13].

Gut microbiota can produce several host biochemicals with known neuromodulatory properties, including serotonin, and, conversely, host serotonin signaling impacts gut microbiota composition. This bidirectional interaction between the gut and brain is associated with the maintenance of host nervous system health. Several recent studies show that there is a strong relation between the regulation of behavior and in the pathophysiology of several mental disorders, including MD and the gut microbiota diversity and composition. Human gut microbiota in MDD is distinct with reduced microbial diversity from that of healthy subjects. Moreover, fecal microbiota transplantation from depression patient to germ free mice resulted in depression-like behaviors [14,15].

Gut microbiota includes complex microbial communities with approximately 10^13^–10^14^ microorganisms. Microorganisms vary from the distal intestine to the large intestine, and, during the average lifespan, several factors impact the gut microbial community, such as age, diet, use of various therapeutics, antibiotics, probiotics, and prebiotics [16,17]. The microbiota plays a vital role in the fermentation and absorption of undigested carbohydrates by interacting with the dietary components and contributing to overall energy homeostasis by collecting and storing energy. Additionally, the intestinal microbiota helps the immune system by promoting immune cells, regulating inflammation, intestinal permeability, glucose and lipid metabolism, and regulating insulin sensitivity [18,19,20,21]. Due to all these effects, a wide range of studies has been carried out based on the interaction between microflora and various human diseases, including cancer, diabetes, and brain abnormalities, many of which focused on how the gut microflora affects mental health [11,14,16,20].

T1D is a common chronic, organ-specific, autoimmune disease that destroys pancreatic β-cells by attaching T-lymphocytes. The incidence of T1D increases universally at a rate of 3–5% per year. Factors that increase the risk of T1D are viral infections, diet, vitamin D deficiency, and the abuse of antibiotics. The dysregulation of the gut microbiome plays a pivotal role in the pathogenesis of T1D, and the microbiome of a T1D patient is defined by a reduced number of short-chain fatty acid (SCFA)-producing bacteria and a deficiency of the lactate-producing bacteria that assist in maintaining intestinal homeostasis. Another substantial issue for individuals who suffer from T1D is major depression and anxiety. Although the use of antidepressants can be a solution to these problems, they can also cause hypoglycemia. In this context, the type of antidepressant used is critical [4,18].

The relationship of the intestinal flora to depression and diabetes and the importance of gut flora for human health is increasingly evident. Studies showed that gut microbial composition varied individually depending on host conditions, and the microbiome can impact the effectiveness of drugs. Therefore, consideration of gut flora when deciding on a drug and during its use is necessary. Herein, with this study, we investigated the effect of reboxetine, which is used to treat major depression and can be safely used for diabetic patients because it does not change the blood glucose level in a few studies [7,22]. To our knowledge, this is the first study evaluating the effect of the antidepressant drug RBX on the gut microbial community in T1D rats.

## 2. Materials and Methods

### 2.1. Drugs

Streptozotocin was purchased from Sigma-Aldrich (St. Louis, MO, USA), Trisodium citrate and citric acid were purchased from Merck (Darmstadt, Germany), and Edronax^®^ (Pfizer, New York, NY, USA) preparation was used for RBX.

### 2.2. Animals

Male Sprague–Dawley rats, aged 12 weeks and weighing 300–350 g, were chosen for the experiments. The animals were maintained in a 12:12 h light–dark cycle (lights on from 8:00 a.m. to 8:00 p.m) and at constant temperature (24 ± 1 °C) in well-ventilated rooms. Rats were allowed free access to water and fed standard pellets. The experimental protocol was performed following the guidelines of the Anadolu University Animal Experiments Local Ethics Committee (2018-57, 4 December 2018).

### 2.3. Induction of Experimental Diabetes

To establish an animal model of diabetes, food was withdrawn overnight, and a single dose of 50 mg/kg streptozotocin (in 0.1 mol/L citric acid buffer, pH = 4.5) was administered intravenously into the tail veins of the animals (i.v.) [23,24,25]. Meanwhile, the rats in the normoglycemic control group received only a citrate buffer at equal volume (i.v.). Following the streptozotocin injection, 5 mmol/L of glucose solution was placed in the rat cages to prevent the risk of hypoglycemic shock [15,26].

### 2.4. Measurement of Blood Glucose Levels

Seventy-two hours after the streptozotocin injection, blood samples were obtained from the tail vein with Accu-Chek Performa Nano^®^ glucose meter (Roche, Basel, Switzerland). Animals with blood glucose levels measuring above 300 mg/dL were considered to be diabetic [23,24].

### 2.5. Experimental Groups

**Figure 1:**
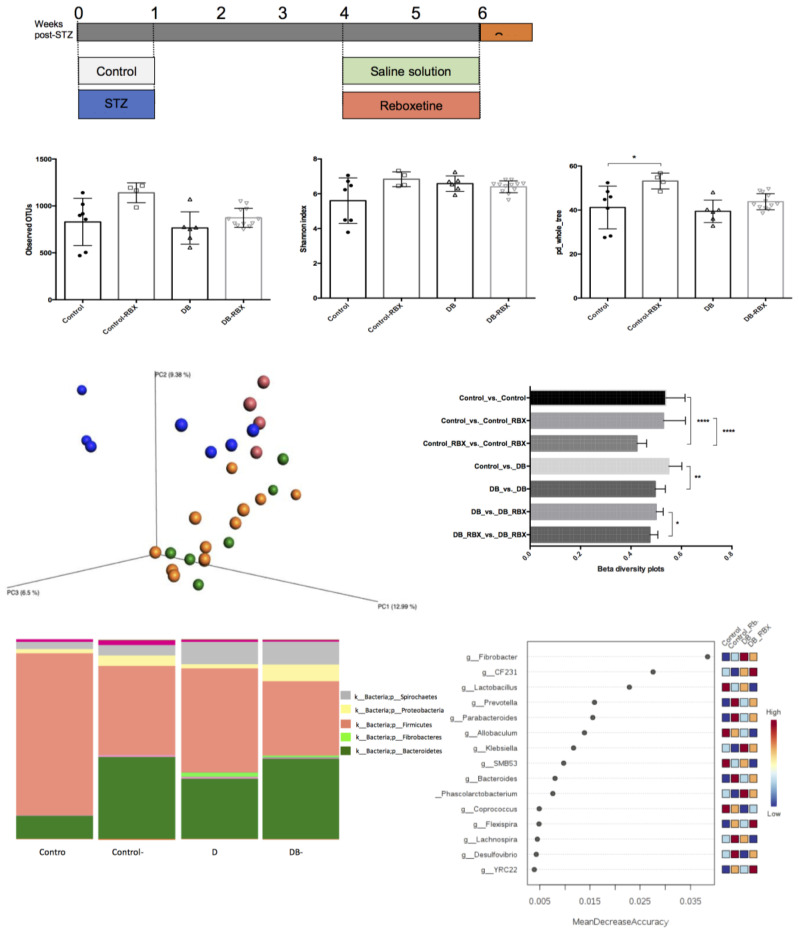
Effect of RBX on intestinal microbial community. (**A**) Study design: Either streptozotocin (STZ) or vehicle was introduced for 1 week. Reboxetine (RBX) was administered at week 4 post-STZ for 14 days. (**B**) Alpha diversity comparisons between the groups (observed OTUs, Shannon index, and phylogenetic diversity). (**C**) Unweighted UniFrac analysis between all groups (*p* = 0.001) and beta diversity pilot distance comparisons within and between groups. (**D**) Relative taxa abundances between all groups at phylum level. (**E**) Random forest analysis of control and diabetic rats. Kruskal–Wallis test with Dunnett’s multiple comparisons test was used for statistical significance. * *p* < 0.05; ** *p* < 0.01; **** *p* < 0.0001.

Animals were randomly divided into four experimental groups (N = 6) housing in separate cages as follows: (i) normoglycemic control group (animals receiving a saline solution); (ii) diabetic control group (animals receiving a saline solution); (iii) RBX-treated normoglycemic groups (animals receiving 8 mg/kg RBX); and (iv) RBX-treated diabetic groups (animals receiving 8 mg/kg RBX) [7,27,28] (Figure 1A).

RBX or saline (controls) administrations were initiated four weeks after the onset of diabetes to develop microbiota changes [21]. RBX was dissolved in saline, all the administrations continued for 14 days via the intragastric route (p.o.), and samples were collected on the 14th day. The RBX dose was determined in preliminary trials by experimenting with two different doses (8 mg/kg vs. 16 mg/kg). Animals were anesthetized and euthanized intraperitoneally with an 8:1 mixture of 2.5% ketamine hydrochloride (Ketalar, New York, NY, USA) and 2% xylazine (Rompun, Bayer, Leverkusen, Germany) [29]. Stool samples were collected under sterile conditions and stored in individual sterile tubes. These tubes were immediately placed in individual containers full of liquid nitrogen and subsequently kept in the freezer (−80 °C) until DNA isolation.

### 2.6. DNA Isolation and Library Preparation

Stool samples were collected after the end of RBX treatment. DNA was isolated according to the instruction of Fast DNA Spin kit (MoBio, Carlsbad, CA, USA). The quality of the DNA was determined in a NanoDropOne spectrophotometer (Thermo Scientific, Waltham, MA, USA), and DNA purity was analyzed by agarose gel electrophoresis. The 16S rRNA gene primers used targeted the V3-V4 region of the gene. Amplicons were prepared in triplicate, pooled, and quantified. Samples were sequenced on the MiSeq sequencing platform (Illumina, San Diego, CA, USA) using a Miseq V3 kit, following standard Illumina sequencing protocols [15].

### 2.7. Microbial Community Analysis

The Quantitative Insights Into Microbial Ecology (QIIME) program 1.90 was employed to analyze data. Sequences were quality filtered and chimeras removed, as previously detailed [30]. Filtered reads were clustered into 97% identity OTUs using the UCLUST program, followed by taxonomy assignment. Alpha diversity was calculated to determine the differences within the microbial community. The phylogenetic tree and abundance tables were used to calculate unweighted and weighted UniFrac β-diversity indices. Relative taxa abundances were also determined.

### 2.8. Statistical Analysis

Significant differences in alpha diversity between experimental groups were determined using a non-parametric *t*-test with 1000 permutations, while differences in β-diversity were tested by permutational MANOVA. Significant differences in relative abundance were assessed using Linear discriminant analysis effect size (LEfSe) with *p*-value < 0.05 and LDA score > 2. A non-parametric Kruskal–Wallis test was utilized, followed by Dunn’s post-hoc test for multiple comparisons.

## 3. Results

### 3.1. Microbial Community Differences between the Groups

Our preliminary experiments on the effect of RBX doses on gut microbiota (8 mg/kg vs. 16 mg/kg) showed that there was no major difference in the effect demonstrated between the different doses (Supplementary Figure S1). Therefore, experiments were performed with an 8 mg/kg dose. A total of 3728 OTUs were determined after filtering the low-abundant OTUs. The mean read count in 29 samples was 26,101. There was no significant difference in observed OTUs and Shannon index between the experimental groups (Figure 1B). However, phylogenetic diversity (pd_whole_tree) was significantly higher in RBX-treated control rats (*p* = 0.02).

Beta diversity analysis revealed significant differences between specific groups indicating the distinct effects of diabetes status and RBX use on microbial community structure. Firstly, we used beta diversity to analyze the effects of diabetes induction in rats. Unweighted UniFrac measurements revealed that diabetic rats have a distinct microbial community structure when compared to the healthy rats, as expected. Another result was that RBX treatment had a significant effect on the microbial community structures of both diabetic and healthy rats (Figure 1C). Weighted UniFrac measurements confirmed the effect of RBX on gut microbial community in both control and diabetic rats. However, no significant effect was found between diabetic and control rats, according to Bonferroni-corrected *p* values (Supplementary Figure S2).

### 3.2. Relative Taxa Abundances in Phylum Level between the Groups

According to relative taxa abundance results, the Fibrobacteres and Spirochaetes phyla were significantly abundant in diabetic rats when compared to controls (*p* < 0.05 for both) (Figure 1D). Interestingly, the Firmicutes/Bacteroidetes ratio was higher in non-diabetic control rats when compared to diabetic rats. In both control and diabetic rats, RBX treatment reduced Firmicutes and increased Bacteroides and Proteobacteria phyla. Moreover, in both diabetic and non-diabetic rats, Prevotellaceae increased, and Lactobacillaceae and Clostridiaceae families decreased with RBX treatment (Supplementary Figure S3).

### 3.3. Differential Taxa in Diabetic and Reboxetine-Treated Rats

Random forest analysis of the top 15 taxa at the genus level revealed that Fibrobacter was the most discriminative feature of diabetes status independent from RBX use. *Lactobacillus* was significantly decreased with RBX treatment and was the most discriminative taxa for the control group. Independent of diabetes status, Prevotella and Parabacteroides had the highest discriminatory power after RBX treatment (Figure 1E).

**Figure 2:**
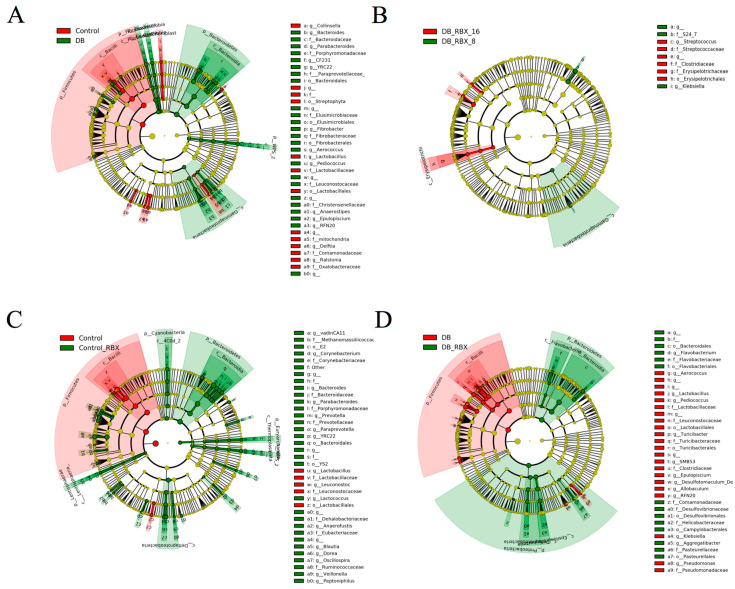
LEfSe cladograms indicating significantly differential taxa. (**A**) Control vs. RBX-treated rats. (**B**) Diabetic vs. RBX-treated diabetic rats. (**C**) Diabetic rats vs. nondiabetic control rats.

LEfSe analysis was performed to identify significantly discriminative taxa between the experimental groups (Figure 2A–C). As expected, diabetic and control rats have several discriminative taxa. According to LDA scores, Bacteroidetes and Fibrobacter were the most discriminative taxa for diabetic rats, whereas bacterial groups, such as *Lactobacillus* and *Bacilli,* were significantly abundant in control rats (Figure 2C).

LEfSe analysis revealed that, in both diabetic rats and control rats, RBX treatment leads to a significant abundance of Bacteroidetes (Figure 2A,B). For the non-diabetic group, the most significantly discriminative taxa after RBX treatment were Bacteroidia, *Prevotella*, Ruminococcaceae, Desulfovibrionaceae, Oscillospora, Bartonellaceae, and *Bacteroides*.

By contrast, *Lactobacillus*, Lactobacillaceae, *Leuconostoc*, and *Lactococcus* diminished with RBX treatment in the control group (Figure 2A). In the diabetic group, RBX treatment led to a significant abundance in Bacteroidetes, Proteobacteria, Campylobacterales, Helicobacteriaceae, and Desulfovibrionaceae. While, in the diabetic RBX-challenged group, *Aerococcus*, *Lactobacillus*, *Pediococcus*, Lactobacillaceae, *Turicibacter*, Clostridiaceae, *Allobaculum*, and *Klebsiella* diminished (Figure 2B).

## 4. Discussion

The prevalence of major depression in people with type-1 diabetes is 2–3 times higher than in non-diabetics. When major depression is associated with diabetes, it causes a decrease in diabetes control due to the increase in diabetes-related complications. Whether this is associated with structural brain anomalies, neurocognitive symptoms and neuroendocrine dysfunctions that occur in parallel in major depression and diabetes require further studies. Antidepressants are a common treatment option for moderate or severe depression. In addition, the presence of side effects of antidepressants, such as postural hypotension, dry mouth, constipation, memory, or cognitive impairment and sexual dysfunction, unfortunately complicates the treatment process, especially in patients with secondary diseases such as diabetes. There is also ongoing debate about the possible effects of antidepressants on blood sugar. This effect is the last desired state for diabetic patients, so it raises question marks in terms of the safe use of antidepressants in these patients. It is critical to ensure that the drugs used by these people to prevent depression do not cause a tertiary deterioration. For this reason, it is important to investigate the effects of these drugs other than those known in diabetic models in terms of safe drug use in diabetic patients [31,32]. Despite remarkable scientific interest in the well-defined effects of various antibiotics [19,33,34] and non-antibiotic drugs [35] across microbial community structure, the impact of antipsychiatry drugs on the gut microbiome has generally been disregarded [36]. In this context, with this study, we investigated the effect of RXB, an antidepressant, on gut flora. Our motivation for choosing reboxetine was because it is an antidepressant with low side effects. Besides, it has been observed that a 4-week treatment with reboxetine (8–12 mg/L) has beneficial effects on metabolic parameters in major depression patients [37]. In schizophrenia patients, the addition of reboxetine to the treatment with olanzapine improved the metabolic and endocrine profiles, leading to a reduction in triglycerides and leptine levels, and an increase in cortisol and DHEA concentration, as well [38] The secondary and most important reason to prefer RBX to evaluate the effect of type-1 diabetes and antidepressant clinical status on gut flora is RBX (2.5 mg/kg/day) is considered as safe for diabetic patients since it does not affect blood glucose and insulin levels in STZ- induced diabetic rats and non-diabetic rats [22]. This finding was also confirmed in our study and no significant difference in the rat plasma glucose levels after RBX treatment (8 and 16 mg/kg for 2 weeks) in diabetic and rats was detected [7].

Considering the previously described antimicrobial consequence of psychotropic drugs, we hypothesized that reboxetine could alter gut microbiome composition. We tested this hypothesis by developing a model of T1D alongside the healthy group of rats for two main reasons. First, clinical studies have demonstrated a two-fold increase in the prevalence of depression in T1D and T2D patients compared to the general population, and second, to investigate the impact of RBX in a readily impaired microbiota caused by a pathology [39].

It should be noted that our diabetes model was designed to mimic T1D with an STZ challenge. Although STZ is a chemotherapeutic and alkylating agent that targets insulin-producing beta cells in the pancreas to mimic T1D phenotype in rats, its partial antimicrobial activity suggests that it may have potential as a suitable agent in microbiota studies. However, Patterson et al. (2015) performed an analysis of the effects of STZ on the gut microbiota of rats, which indicated that the changes in gut microbiota composition were likely to be a result of T1D onset and progression rather than its antimicrobial activity [40].

T1D findings revealed that the Firmicutes/Bacteroidetes ratio was lower in our streptozotocin-induced model. This finding is compatible with previous literature and confirmed the successful induction of the diabetes model in our experiment. The elevated Firmicutes/Bacteroidetes ratio has been associated with an increased capacity to harvest energy from the diet and is related to the obesity phenotype [41]. Since weight loss is an expected consequence of streptozotocin-induced diabetes, the decreased Firmicutes/Bacteroidetes ratio in diabetic rats might be due to a change in body weight. Drug administrations, including antidepressants, have differential effects on the Firmicutes/Bacteroidetes ratio. In our study, the Firmicutes/Bacteroidetes ratio decreased in both healthy and diabetic rats after RBX administration [33,40,42,43]. Interestingly, research by Cusotto et al. (2019) showed that, by investigating the effects of various psychotropics on gut microbiota, the Bacteroidetes levels significantly decreased after administering the antipsychotic drugs lithium, valproate, and aripiprazole [36]. Bacteroidetes levels increased with the administration of antidepressants, such as escitalopram (SSRI), venlafaxine (SNRI), and fluoxetine (SSRI) [36].

In addition to Bacteroidetes, Proteobacteria also became abundant after RBX treatment in both groups. The abundance of Proteobacteria might indicate endotoxemia, which is an inflammatory condition induced by LPS. As a proinflammatory molecule, LPS causes an increase in fat storage in the host. Previous studies showed a positive correlation between LPS and many diseases, especially with type 2 diabetes (T2D) associated with high plasma glucose levels [44,45]. Inflammation and unbalanced microbial groups are possible in diabetic subjects because most of the metabolism-associated diseases are associated with increased plasma lipopolysaccharide concentration due to increased intestinal permeability, potentially prompting a rise in proinflammatory cytokines [46]. However, it is interesting that *Klebsiella*, another enteric bacterium that is a sign of LPS, was found lower after RBX administration in our findings. Although *Klebsiella* is generally known for causing lung infections, studies have shown that it can also trigger intestinal inflammation [47]. In the STZ-induced diabetes model, *Klebsiella* was predominant and could be considered a signature of T2D. After insulin treatment in T1D rats, the abundance of *Klebsiella* decreased [48]. As previously argued, the exact role of *Klebsiella* is not clear in this scenario, whether it affects diabetes onset or not. If so, the effects of RBX on *Klebsiella* are significant.

Another notable effect of RBX, which was frequent for both control and diabetic rats, was a bloom in Bacteroidales and a drop in *Lactobacillus* abundance. Considering that *Lactobacillus* levels were already diminished in diabetes-induced rats, RBX administration may have arguably had a disruptive effect on *Lactobacillus*, particularly in diabetic subjects. *Lactobacillus* is known for its beneficial effects on the intestinal system and plays a vital role in the degradation of complex polysaccharides to SCFAs [49,50]. Moreover, when considering the bi-directional relationship between the central nervous system and gut, we must pay attention to decreased *Lactobacillus* level, because *Lactobacillus* reduces anxiety and depressive-like behaviors due to their indispensable role in producing gamma-aminobutyric acid (GABA) [51]. The study by Cusotto et al. (2019) showed the in-vitro antimicrobial activity of various psychotropic medications on *Lactobacillus rhamnosus* [36]. Our present results support this finding by confirming the in-vivo effect on *Lactobacillus* spp. This research underscores the importance of evaluating the ramifications of psychotropic medications against beneficial bacteria in clinical settings.

Bacteroidetes are the main producers of acetate and propionate, whereas butyrate production is predominantly mediated by members of Firmicutes [52]. While SCFA production is not solely associated with a specific bacterial taxon [53], we may expect lower levels of butyrate production in RBX-treated group in accordance with the decreased Firmicutes. Given the importance of butyrate in maintaining intestinal barrier integrity and suppressing inflammation, [52], the lack of a butyrogenic effect may lead to perturbations in immunomodulatory functions and increased permeability in RBX-treated groups. A higher pH in colon due to lower SCFA levels in RBX-treated groups may also contribute to gut microbial alterations, including decreased levels of *Lactobacillus* and a bloom in Bacteroidetes in our RBX groups. While the correlations between butyrate levels and *Lactobacillus* abundance has been previously reported [54], this finding lacks causality, and may possibly be explained by complex cross-feeding mechanisms between *Lactobacillus* spp. and butyrate producers together with the pH levels. *Lactobacillus* can also mediate regulation of substrate transporters, such as monocarboxylate transporter 1 (MCT1), which facilitate the SCFA absorption by colonocytes [55]. Considering that more than 90% of the SCFAs in colon lumen are absorbed, the diminished levels of *Lactobacillus* in RBX groups may also lead to less absorption of the SCFAs [56].

Significant increases were evident in the Desulfovibrionaceae and Helicobacteraceae phylum after RBX administration in the diabetes model. An earlier report showed that the sulfate-reducing bacterium Desulfovibrionaceae is associated with many diseases, such as obesity, inflammatory bowel disease, and T2D [57,58]. In addition, Helicobacteraceae, which produce ammonia during inflammation, is connected with intestinal inflammation [59,60]. The presence of Helicobacteraceae and *Desulfovibrio* is deemed discriminative for active colitis [61]. Moreover, as the major genus of Helicobacteraceae, *Helicobacter* has previously been identified as being highly coated with IgA, along with the *Prevotellaceae* family, indicating their inflammation-driving capability in the gut [62]. Indeed, *Prevotellaceae* was found significantly more abundant after RBX exposure in our control group. We believe these results provide evidence that RBX may lead to an increased inflammation-driving capacity in gut microbiota. Moreover, RBX has no anti-inflammatory properties that might reverse inflammation [62]. Thus, RBX may cause a low-level inflammatory effect in the gut.

Mental disorders are associated with an upregulation of inflammatory molecules. Some antidepressants have the ability to inhibit the release of pro-inflammatory cytokines and trigger the production of anti-inflammatory cytokines. However, the mechanism behind this is still unknown [63,64]. Dong et al. (2016) showed that escitalopram (3 or 10 mg/kg) significantly increased the serum levels of the anti-inflammatory cytokine interleukin-10 (IL-10) by a single administration of LPS. In contrast, pretreatment with R-citalopram (10 mg/kg, i.p.) or reboxetine (10 mg/kg, i.p.) did not affect the alterations in serum levels of TNF-α and IL-10 after LPS administration [59,65].

We acknowledge the following limitations of our present study. First, we did not test the antimicrobial activity spectrum for RBX. However, one study by Cussotto et al. (2019) demonstrated an antimicrobial effect of some antidepressants on *Lactobacillus rhamnosus* 6118 and *Escherichia coli* APC 105 [36]. While the antimicrobial activity might be concentration-dependent, the consistent decrease in *Lactobacillus* genus in both control and diabetic rats could be due to its antimicrobial activity. Second, the longitudinal analysis might be a better approach to understanding specific microbial changes over time, focusing primarily on the status of *Lactobacillus.*

## 5. Conclusions

Considering all study results, we conclude that RBX use causes significant changes in specific taxa and leads to a propensity towards an altered gut microbiome with increased inflammatory capacity, as discussed above. This effect must be considered, especially in individuals with pre-existing conditions such as diabetes. The use of probiotics is advisable to prevent an undesirable decrease in *Lactobacillus.*

## Data Availability

The 16S sequencing data that support the findings have been deposited in QIITA (https://qiita.ucsd.edu/) with the identifier 13160 (https://qiita.ucsd.edu/study/description/13160).

## Supplementary Materials

The following are available online at https://www.mdpi.com/article/10.3390/microorganisms9091948/s1, Figure S1: LeFSe analysis comparing different doses of RBX on microbial taxa (D16: RBX dose 16 mg/kg, DB8: RBX dose 8 mg/kg), Figure S2: Relative taxa abundances between groups in family level, Figure S3: Relative taxa abundances between groups in family level.
