# Supplementary material for: Gut Microbiome Alteration after Reboxetine Administration in Type-1 Diabetic Rats
Source: Microorganisms. 2021 Sep 14;9(9):1948. doi: 10.3390/microorganisms9091948 (PMC8465486; doi:10.3390/microorganisms9091948)
Supplement: Supplementary file 1 [file microorganisms-09-01948-s001.zip › supp data 3 2/supp3.pptx]

## Slide 1
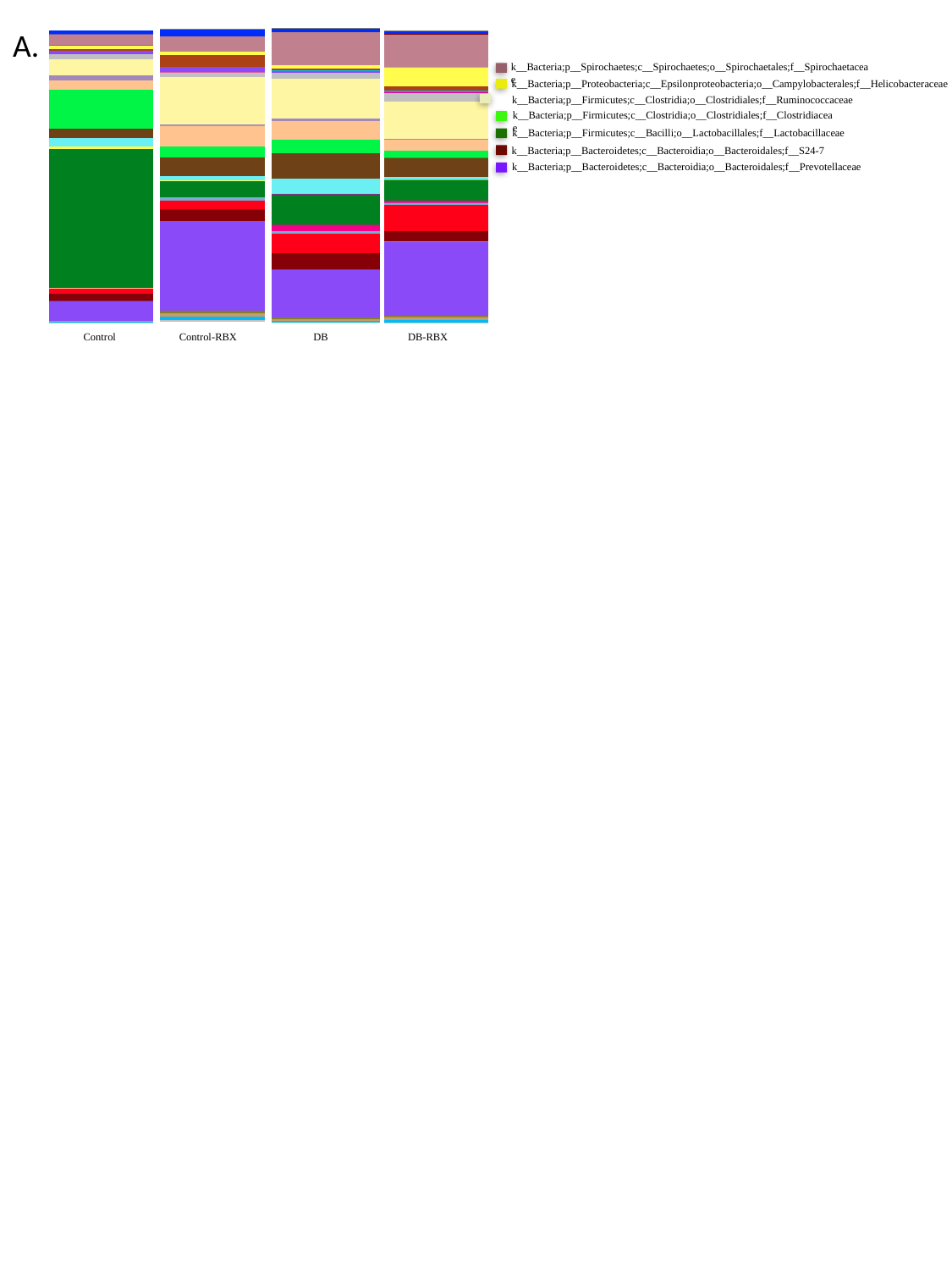

A.
k__Bacteria;p__Spirochaetes;c__Spirochaetes;o__Spirochaetales;f__Spirochaetaceae
k__Bacteria;p__Proteobacteria;c__Epsilonproteobacteria;o__Campylobacterales;f__Helicobacteraceae
k__Bacteria;p__Firmicutes;c__Clostridia;o__Clostridiales;f__Ruminococcaceae
k__Bacteria;p__Firmicutes;c__Clostridia;o__Clostridiales;f__Clostridiaceae
k__Bacteria;p__Firmicutes;c__Bacilli;o__Lactobacillales;f__Lactobacillaceae
k__Bacteria;p__Bacteroidetes;c__Bacteroidia;o__Bacteroidales;f__S24-7
k__Bacteria;p__Bacteroidetes;c__Bacteroidia;o__Bacteroidales;f__Prevotellaceae
DB-RBX
Control
Control-RBX
DB
