# Supplementary figures and images for: Gut Microbiome Alteration after Reboxetine Administration in Type-1 Diabetic Rats
Source: Microorganisms. 2021 Sep 14;9(9):1948. doi: 10.3390/microorganisms9091948 (PMC8465486; doi:10.3390/microorganisms9091948)

## Slide 1
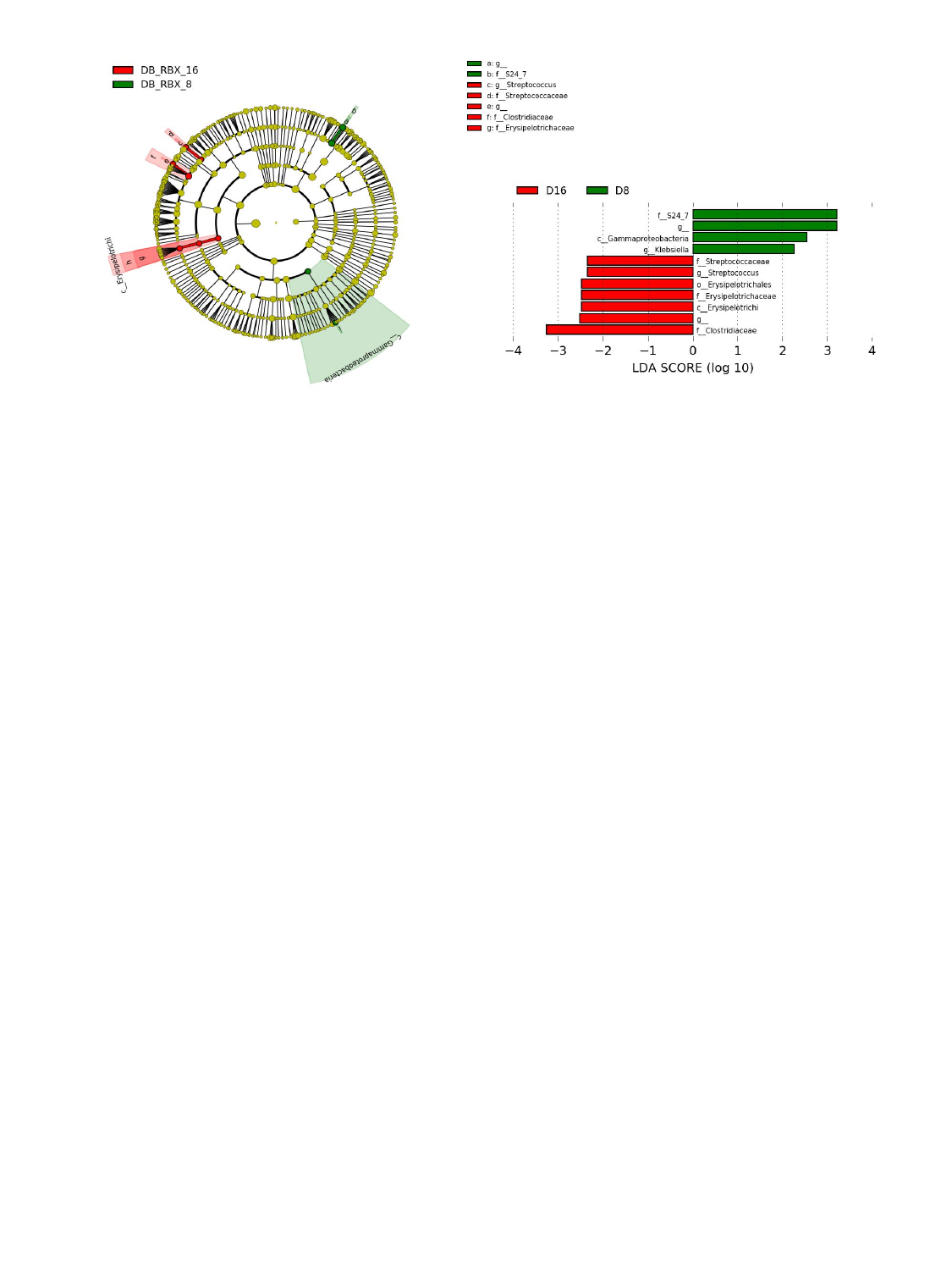

Supplement: Supplementary file 1 [file microorganisms-09-01948-s001.zip › supp data 3 2/supp1.pptx]

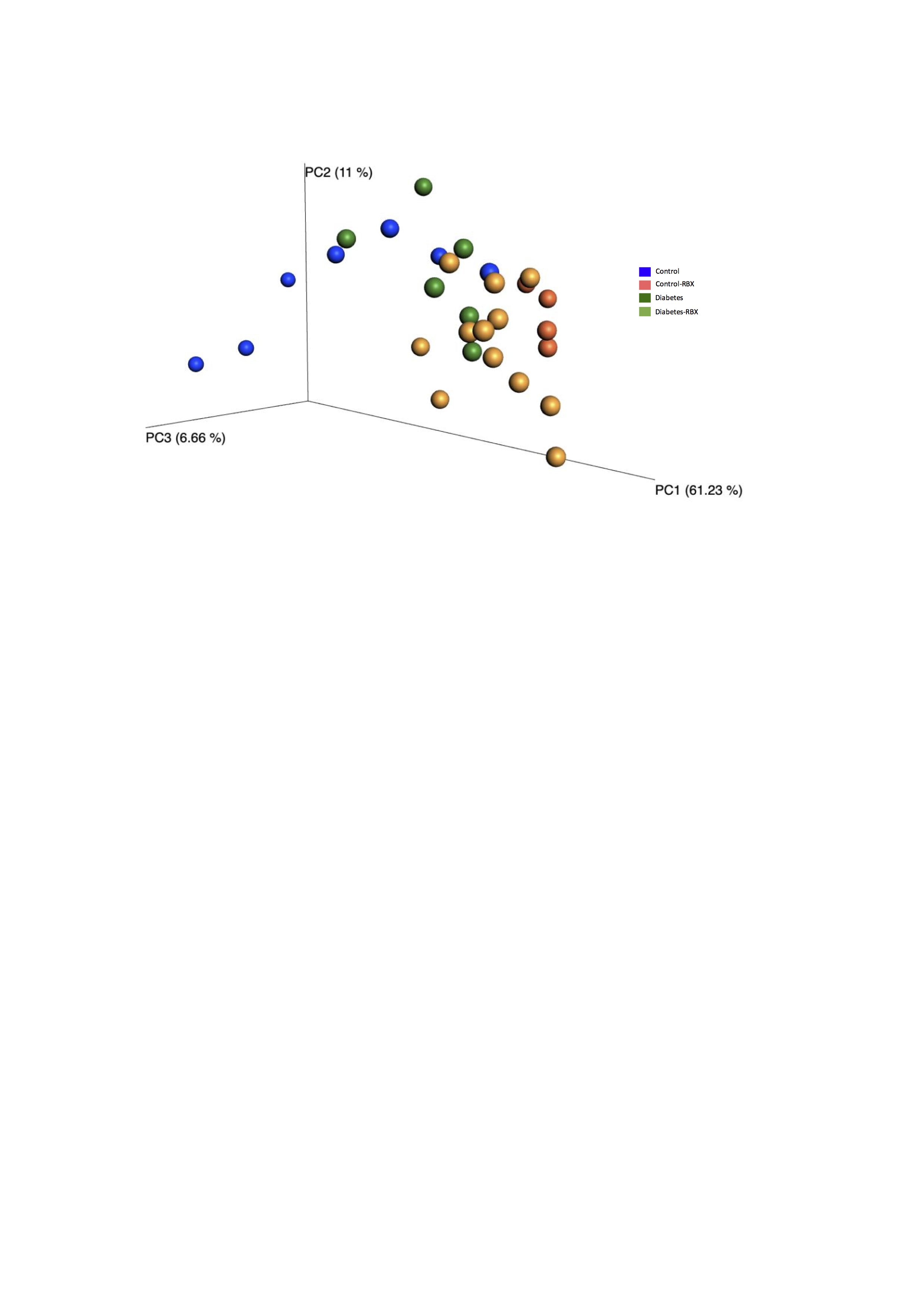

Supplement: Supplementary file 1 [file microorganisms-09-01948-s001.zip › supp data 3 2/suppl fig 2.png]
